# Tumor microenvironment-responsive multifunctional peptide coated ultrasmall gold nanoparticles and their application in cancer radiotherapy

**DOI:** 10.7150/thno.45017

**Published:** 2020-04-06

**Authors:** Yuan Ding, Zhongquan Sun, Zongrui Tong, Sitong Zhang, Jie Min, Qianhui Xu, Liuzhi Zhou, Zhengwei Mao, Haibing Xia, Weilin Wang

**Affiliations:** 1 Department of Hepatobiliary and Pancreatic Surgery, the Second Affiliated Hospital, Zhejiang University School of Medicine, Hangzhou, Zhejiang 310009; 2 MOE Key Laboratory of Macromolecular Synthesis and Functionalization, Department of Polymer Science and Engineering, Zhejiang University, Hangzhou 310027, China; 3 Key Laboratory of Precision Diagnosis and Treatment for Hepatobiliary and Pancreatic Tumor of Zhejiang Province, Hangzhou, Zhejiang 310009; 4 Research Center of Diagnosis and Treatment Technology for Hepatocellular Carcinoma of Zhejiang Province, Clinical Research Center of Hepatobiliary and Pancreatic Diseases of Zhejiang Province, Hangzhou, Zhejiang 310009; 5 Clinical Medicine Innovation Center of Precision Diagnosis and Treatment for Hepatobiliary and Pancreatic Disease of Zhejiang University, Hangzhou, Zhejiang; 6 Clinical Research Center of Hepatobiliary and Pancreatic Diseases of Zhejiang Province, Hangzhou, Zhejiang 310009; 7 State Key Laboratory of Crystal Materials, Shandong University, Jinan 250100; 8 Department of Hepatobiliary and Pancreatic Surgery, the First Hospital of Jiaxing, Jiaxing, Zhejiang 330440

**Keywords:** enzyme-responsive, Tat peptide, zwitterionic peptide, ultrasmall gold nanoparticles, cancer radiotherapy

## Abstract

Two important features are required for promising radiosensitizers: one is selective tumor cell targeting to enhance the therapeutic outcome via lethal DNA damage and the other is rapid clearance to enable excellent biocompatibility for potential clinical application. Herein, ultrasmall gold nanoparticles (Au NPs) with diameter smaller than 5 nm were prepared and covered with a multifunctional peptide to endow them with selective tumor cell uptake capability. Combined with X-ray irradiation, the responsive Au NPs demonstrated superior radio-sensitizing toxicity and rapid renal clearance *in vivo*.

**Methods**: A responsive peptide (Tat-R-EK) consists of three build blocks were used: a cell and even nuclear penetrating block derived from human immunodeficiency virus-1 transactivator of transcription protein (Tat), an cathepsin B cleavable linker, and a zwitterionic antifouling block. Ultrasmall Au NPs were prepared and then covered by the peptide via the Au-S bonds between gold and thiol groups from cysteine. The morphology, colloidal stability and the responsiveness of obtained Au@Tat-R-EK NPs were studied using transmittance electron microscopy and dynamic laser scattering. The selective cancer cell uptake and accumulation of Au@Tat-R-EK NPs in cancer tissue were studied via ICP-MS *in vitro* and *in vivo*, respectively. The cytotoxicity of Au@Tat-R-EK NPs on HepG2 cancer cells was evaluated in terms of cell viability, DNA damage, intracellular reactive oxygen species generation, and apoptosis analysis. Finally, the biocompatibility and tumor destruction ability against orthotopic LM3 liver cancers were verified *in vivo*.

**Results**: Multifunctional peptide modified ultrasmall Au NPs were successfully prepared. The Au NPs exhibited enough colloidal stability and cathepsin B-responsive surface change, leading to selectively uptake by cancer cells *in vitro* and accumulation to tumor sites* in vivo*. Combined with X-ray irradiation, the responsive Au NPs demonstrated superior radio-sensitizing cytotoxicity *in vitro* and therapeutic outcome on mouse liver cancer *in vivo*. The ultrasmall size enables rapid clearance of the Au NPs, guarantees the biocompatibility *in vivo* for potential clinical applications.

**Conclusion**: Some obstacles faced by the Au NPs-based radiotherapy, such as short circulation half-life, non-specific distribution, slow clearance and low radio-sensitizing effect, were effective solved through rational design of the smart nanomedicine. This work provides new insight in designing tumor microenvironment-responsive nanomedicine in cancer radiotherapy.

## Introduction

Radiation therapy is one of the most widely used non-surgical treatments for cancer patients in clinic, due to its high tissue penetration 1, 2. But the application of high-energy radiation, usually X-rays, can destroy DNA double strands and thus need to be restricted to cancer cells/tissues to reduce the damage to the surrounding healthy tissues 3, 4. Therefore, one strategy is to deliver agents which are able to interact with X rays (radiosensitizers) into desired cells/tissues to localize the radiation dosage 5-8.

With the development of nanotechnology, nanoparticles have advantage over conventional molecular radiosensitizers because nanoparticles can be designed to enable the preferential accumulation into tumor tissues due to the enhanced permeability and retention (EPR) effect 9-11. Many high atomic number metal-based nanomaterials including gold 12, 13, bismuth 14-16, gadolinium 17, hafnium 8, 18 and so on, have been extensively studied as radiosensitizers. Among them, gold nanoparticles (Au NPs) have been widely studied because they have intrinsic radiosensitive capability due to high atomic number, excellent biocompatibility compared to other high atomic number materials 19-21. Additional advantages of Au NPs are that they can be used as contrast for imagining guided therapy 22, 23 and are suitable for combinational therapy with nanocarriers of other drugs 24, 25.

Although the radiosensitivity of Au NPs are widely acknowledged, one challenge is still remaining for their clinical translation. An ideal radiosensitizer shall simultaneously have rapid clearance from the body to minimize the long-term toxicity, and high internalization in tumor tissue/cell and even cell nucleus to achieve a therapeutic relevant concentration 26-29. Ultrasmall size (hydrodynamic diameter < 5.5 nm) of nanomaterials shall be required to achieve rapid renal clearance because they can pass through the glomerular capillary wall in kidney 30-33. Besides, the ultrasmall size also has contribution to deeper tumor penetration and larger surface area to volume ratio, which are beneficial for enhancing radiosensitive outcome 12, 34, 35. For example, Zhang et al. 12 prepared gold clusters comprised of only a few gold atoms and ultrasmall size, coated with a hydrophilic glutathione tripeptide shell. The obtained gold nanoclusters could escape from the reticulo-endothelial system (RES) absorption and passively accumulate to tumor tissues, achieved promising radiotherapy sensitization. The efficient renal clearance of the ultrasmall-size gold clusters could prevent the accumulation in liver and reduce the toxicity generated by radiotherapy.

However, only a hydrophilic inert coating is not favorable for the accumulation of ultrasmall NPs inside cell nucleus, where DNA damage happens during the radiotherapy. It is appealing to enable the active-targeting of ultrasmall NPs to cancer cell nucleus via a sophisticate surface design 36-40. To fulfill this purpose, a programmed multifunctional surface coating is required: it can firstly enable the hydrophilic and stealthy nature of the NPs for efficient blood circulation and tumor penetration; it then turn into cell penetrating and nucleus targeting after the NPs reached tumor cells to enhance the cell uptake and nucleus accumulation.

To design such a cascade delivery coating, three components are required: a cancer cell penetrating and cell nucleus targeting moiety shall be immobilized on the NPs as the inner layer, an antifouling coating shall be constructed as the outer layer, and a tumor microenvironment responsive linkage shall be introduced. The outer shielding coating could be easily detached in tumor microenvironment to expose the cell penetrating peptides and activate cell internalization and subsequent nuclear targeting.

Many different materials, such as lipids, polysaccharides, polymers and their combinations, can be used to construct responsive coating on Au NPs. Among them, peptides have unique advantages including tailor-designed sequence and biological functionality, excellent biocompatibility and biodegradability, and easy chemical modification and synthesis 41, 42. It is extremely appealing to use peptide to design responsive coating because we can easily design and prepare a multifunctional peptide based on different building blocks via solid phase peptide synthesis (SPPS).

**Figure 1:**
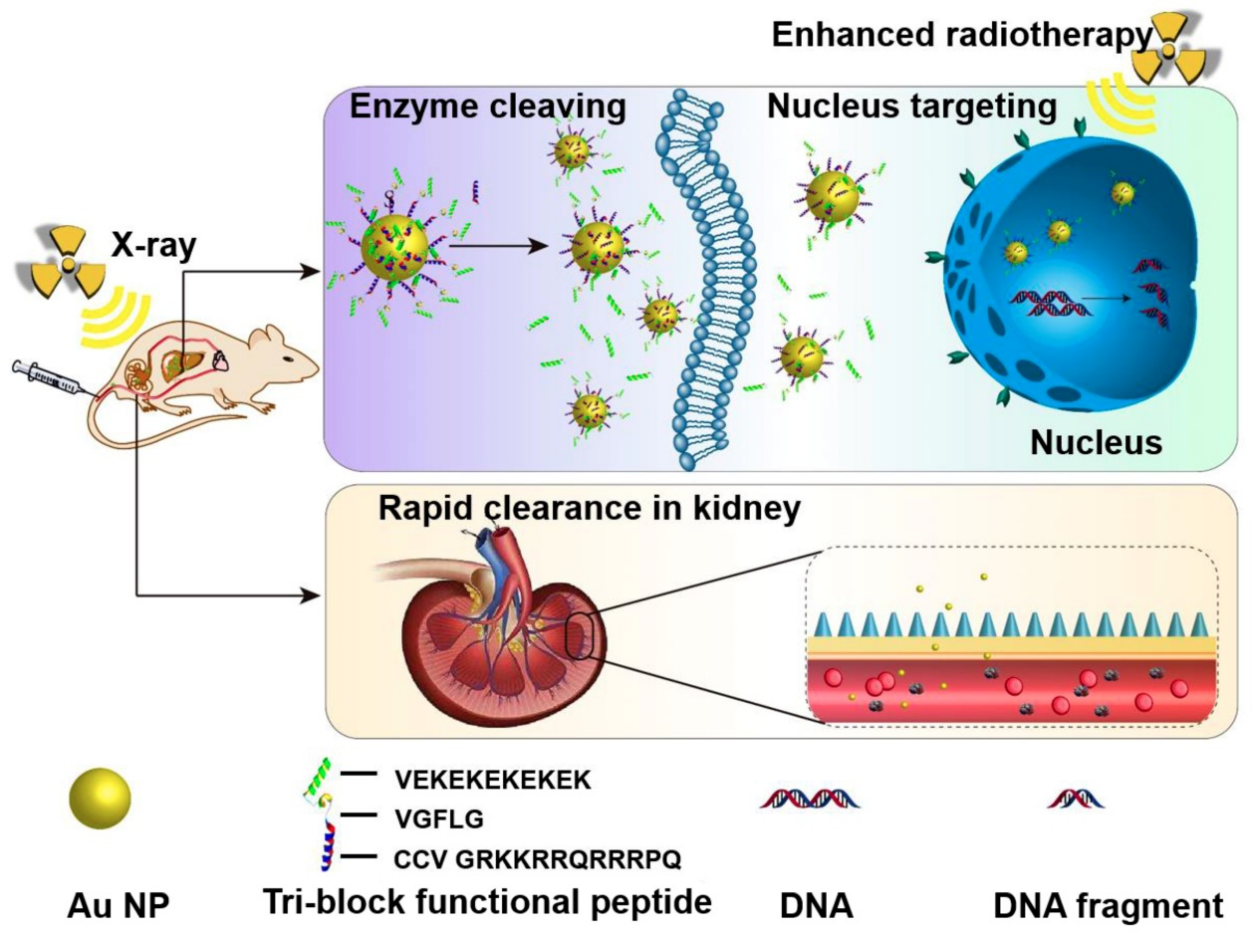
Schematic illustration of the accumulation in tumor tissues and cell nuclei for enhanced radiotherapy *in vivo*, and the rapid clearance via kidney tri-block functional peptides coated ultrasmall Au NPs.

In current study, a powerful cell penetrating and nuclear targeting peptide sequence (GRKKRRQRRRPQ, known as Tat peptide) 43, 44, derived from human immunodeficiency virus-1 (HIV-1) transactivator of transcription protein, was selected to conjugated on Au NPs via modification with two cysteine molecules. A peptide sequence (GFLG), which is known to be cleaved by overexpressed cathepsin B in the microenvironment of many different tumors 45-47, was used to link the Tat peptide and the antifouling peptide sequence. A zwitterionic peptide sequence consisting of alternative glutamic Acid (E) and lysine (K) with great antifouling property *in vivo* 48-50 was adopted as the outside shielding layer to cover the Au NPs, enabling the stable blood circulation property and passive tumor accumulation ability (Figure 1).

The obtained ultrasmall Au NPs showed enough colloidal stability and cathepsin B-responsive surface change, leading to selectively uptake by cancer cells *in vitro* and accumulation to tumor sites *in vivo*. Combined with X-ray irradiation, the responsive Au NPs demonstrated superior radio-sensitizing cytotoxicity *in vitro* and therapeutic outcome on mouse liver cancer *in vivo*. The ultrasmall size enables rapid clearance of the Au NPs, guarantees the biocompatibility* in vivo* for potential clinical applications.

## Methods

### Chemicals

Hydrogen tetrachloroaurate hydrate (HAuCl_4_·4H_2_O), sodium citrate, and tannic acid were obtained from Sinopharm Chemical Reagent Co., Ltd., China. Methylthiazoletatrezolium (MTT), 4',6-diamidino-2-phenylindole (DAPI), human recombinant cathepsin B, and GM6001 (a cathepsin B inhibitor) were purchased from Sigma-Aldrich. Cathepsin B-responsive tri-block peptide (CCVGRKKRRQRRRPQVGFLGVEKEKEKEKEK) and non-responsive peptide (CCVGRKKRRQRRRPQVGLGFVEKEKEKEKEK) were purchased from Synpeptides Co., Ltd., Shanghai, China. The micro-bicinchoninic acid (MicroBCA) protein assay kit and 2',7'-dichlorodihydrofluorescein diacetate (DCFH-DA) cellular reactive oxygen species assay kit were purchased from Beyotime Biotechnology Inc., Nantong, China. Dulbecco's modified eagle media (DMEM) was purchased from Gibco, USA. Fetal bovine serum (FBS) was purchased from Sijiqing Co., Ltd., Hangzhou, China. Cell apoptosis detection kit was obtained from BD Biosciences. Other reagents were analytical grade and used as received. Water purified by a Milli-Q system was used in all the experiments.

### Preparation and characterization of responsive ultrasmall Au NPs

Ultrasmall Au NPs were synthesized according to a previously reported method with slight modification 51. In brief, 45 mL water, 5 mL sodium citrate solution (66 mM) and 0.1 mL tannic acid solution (2.5 mM) were mixed in a 100 mL flask and heated to 70 ^o^C. 1 mL HAuCl_4_ (25 mM) solution was added and kept at 70 ^o^C for about 9 min. After the solution turned into purple color, the reaction was stopped by rapid cooling. The obtained ultrasmall Au NPs solution was purified by dialysis against plenty of water for 8 h at 4 ^o^C, exchanged with fresh water every hour. The Au NPs were concentrated (4000× g, 20 min) using an Amicon Ultra-15 centrifugal filter unit (Merck-Millipore, USA). The concentration of Au NPs was quantified by inductively coupled plasma mass spectrometry (ICP-MS, Xseries II, Thermo Scientific, USA).

As-prepared ultrasmall Au NPs were immediately used for ligand exchange. Briefly, 0.1 mg/mL Au NPs were mixed with peptides at room temperature for 24 h with gentle shaking. Au@Tat-R-EK and Au@Tat-I-EK are the terms used to represent the ultrasmall Au NPs decorated with cathepsin B-responsive peptide and cathepsin B non-responsive peptide, respectively. After three times' washing using the Amicon Ultra-15 centrifugal filter unit, the samples were concentrated and stored at 4 ^o^C. The contents of Au and peptide were confirmed by ICP-MS and microBCA assay, respectively.

The morphologies of ultrasmall Au NPs were recorded under transmission electron microscope (TEM, JEM 2100F, JEOL, Japan). The hydrodynamic diameter and surface zeta potential of ultrasmall Au NPs were characterized by dynamic light scattering (DLS) on a Zetasizer 3000 (Malvern, USA). The spectra of ultrasmall Au NPs were recorded by UV-vis spectroscopy (Shimadzu UV2550, Japan).

The ultrasmall Au@Tat-R-EK NPs were treated with 1 μg/mL cathepsin B in phosphate buffer (PBS, pH 7.4) at 37 ^o^C for 4 h. Afterwards, their UV-vis spectra, hydrodynamic diameter and surface zeta potential were characterized to verify the responsiveness of the peptide coating.

### Cell uptake of ultrasmall Au NPs

Human liver cancer LM3 cells and luciferase transfected LM3 cells were obtained from the Cell Bank of the Chinese Academy of Sciences (Shanghai, China). The cells were maintained in DMEM supplemented with 10% FBS, 100 μg/mL streptomycin, and 100 U/mL penicillin.

To verify the responsive cell penetrating ability of ultrasmall Au NPs, LM3 cells were seeded in a 6-well plate (Corning) at a density of 3.0×10^5^ cells/well and incubated overnight for cell spreading. Fresh medium containing Au@Tat-R-EK or Au@Tat-I-EK NPs (20 μg/mL) were used to incubated cells. At pre-determined time points, the cells were washed with fresh PBS to remove unanchored Au NPs, detached by trypsin treatment and then collected by centrifugation. Half of the cells were counted on a Neubauer chamber to get cell number. The other half of the cells were treated in aqua regia and then diluted 1000 times to measure the cellular gold concentration via ICP-MS. The cells treated with NPs-free medium were used as the control. The cells treated with 1 μg/mL cathepsin B or 20 nM cathepsin B inhibitor (GM6001) were also used to study the cellular uptake pattern of Au@Tat-R-EK NPs with different coatings, respectively.

### Radiosensitivity *in vitro*

To test the radiosensitive property of ultrasmall Au NPs, LM3 cells were seeded in a 96-well plate at a density of 1.0×10^4^ cells/well and incubated overnight for cell spreading. After incubation with media containing Au@Tat-R-EK or Au@Tat-I-EK NPs (5, 10, 20 40 μg/mL respectively) for 24 h, the cells were washed with fresh PBS and then irradiated with X-ray (dosage: 4 Gy). The cell viability was accessed after another 12 h incubation, using the MTT assay, where untreated cells were used as negative control (denote as 100% viability).

### Determination of the Percentage of Apoptotic Cells

LM3 cells were seeded in 6-well cell culture plates (2.0×10^5^ cells/well). After 12 h incubation, the medium was replaced by fresh growth medium containing Au@Tat-R-EK or Au@Tat-I-EK NPs (20 μg/mL). After 24 h incubation, the cells were washed with PBS and treated with X-ray (4 Gy). After another 12 h incubation, the cells were harvested with EDTA-free trypsin (0.25%). Then the cells were stained by FITC labeled annexin-V and propidium iodide (PI) according to the manufacturer's protocol. Flow cytometry (FACS caliber, BD) was used to analyze the percentage of cells under different apoptotic stage. The untreated cells were utilized as the control.

### DNA double-strand breakage

LM3 cells were seeded into 35 mm dishes with glass bottom at a density of 2.0×10^5^ cells/well. After 24 h incubation with Au@Tat-R-EK or Au@Tat-I-EK NPs (20 μg/mL) and then treated with X-ray (4 Gy), the cells were fixed with 4% paraformaldehyde and then were permeabilized by Triton X-100 (0.2%) for 10 min. The cells were immunostained with anti-γ-H2AX antibody (ab81299, Abcam) at 4 °C overnight. The cells were washed with PBS and incubated with the secondary antibody (FITC labeled) for 1 h at 37 °C. 100 ng/mL of DAPI was added to stain the cell nuclei and the cells were observed under a confocal laser microscopy (LSM710, Zeiss, Germany).

### Intracellular ROS Detection

DCFH-DA was employed as a fluorescent reactive oxygen species (ROS) probe to indicate Au NPs sensitized X-ray-induced oxidative stress. The non-fluorescent DCFH-DA can cross the cell membranes. It is deacetylated by esterases inside cells, and then oxidized to the fluorescent 2',7'-dichlorfluorescein (DCF) by ROS. LM3 cells were seeded at a density of 5.0×10^4^ cells per well on a 24-well plate, and were cultured for 12 h. The cells were treated with Au@Tat-R-EK or Au@Tat-I-EK NPs (20 μg/mL) for 24 h, and were then washed with PBS and treated with X-ray (4 Gy). DCFH-DA (25 µM) was added to incubated cells for another 4 h. Then, the cells were washed with PBS three times and collected by trypsin treatment. The ratio of fluorescent (ROS up) cells were analysed by flowcytometry.

### Biocompatibility *in vivo*

Animal experiments were performed according to Guidelines of Animal Care and Use, Zhejiang University. Healthy male nude mice (6-8 week old) were purchased from the animal center of Zhejiang Academy of Medical Sciences, and were cultured for at least 3 days in the pathogen-free animal house under 12 h light/dark cycles at 24 °C. To evaluate the biocompatibility of ultrasmall Au NPs *in vivo*, BALB/c mice were intravenously administrated with ultrasmall Au NPs at a dose of 50 mg/kg. The mice injected with PBS were used as the control group. One day after injection, the blood samples were collected for complete blood panel analysis and serum biochemistry test. Serum interleukin-6 (IL-6) and tumor necrosis factor alpha (TNF-α) levels were quantified by the ELISA assay (Abcam, UK). One day post injection, the mice were sacrificed to harvest major organs (including heart, liver, lung, and kidney) for hematoxylin and eosin (H&E) staining and histological analysis.

### Orthotopic transplanted liver tumor model

The mice were anesthetized by intraperitoneal injection of 1% pentobarbital, and their abdominal cavity was cut open along the midline of the abdomen to expose the left lobe of the liver. Orthotopic transplanted liver tumors were established by injection of 25 μL luciferase transfected LM3 cells suspension (1×10^6^ cells in Matrigel) into the left lobe of the liver. The mice were kept in a warm incubator after the abdominal cavity was sutured up and disinfected using povidone-iodine. About 10 days later, the mice were used for the following experiments, where orthotopic transplanted liver tumors developed to about 6-7 mm in diameter.

### Clearance kinetics and tumor accumulation of ultrasmall Au NPs *in vivo*

Male nude mice bearing orthotopic LM3 transplanted liver tumors were used (n = 4). 200 μL of solution was injected via the tail vein in each mouse. The gold concentration in blood was measured via ICP-MS assay at different time points post injection 52. The urea and feces were collected separately using metabolism cages at different time points. After completely dried, they were treated in aqua regia to dissolve Au NPs. The gold content was then quantified by ICP-MS 53.

The accumulation of ultrasmall Au NPs in tumor and major organs was analyzed at 24 h post injection. Tumors were dissected from liver, weighed, and lyophilized. The dried tumor tissues and major organs were treated with aqua regia, and their gold content was analyzed by ICP-MS after dilution.

### Radiotherapy *in vivo*

The orthotopic transplanted luciferase-LM3 liver tumors mice were divided into five groups (n = 5) and were i.v. injected with (1) 200 μL PBS, (2) 200 μL PBS + X-ray, (3) 200 μL PBS containing Au@Tat-R-EK NPs, (4) 200 μL PBS containing Au@Tat-I-EK NPs+ X-ray, and (5) 200 μL PBS containing Au@Tat-R-EK NPs+ X-ray. The dosage of ultrasmall Au NPs were fixed to 25 mg/kg. The dosage of X-ray is 6 Gy.

Non-invasive monitoring of tumor progression was followed by scanning mice with the IVIS Spectrum-bioluminescent and fluorescent imaging system (IVIS, Caliper Life Sciences, USA). 15 minutes before imaging, 150 μL of D-luciferin (30 mg/mL, Perkin Elmer) in PBS was intraperitoneally injected into each mouse. Whole-animal imaging was taken every 7 days after treatment.

The mice were sacrificed 14 days after treatment and the left lobe of the liver which contains tumors was collected for photograph.

The same amount of LM3-bearing mice (5 groups and each group has 5 mice) were used to draw the survival curve.

### Tumor histological analysis

The tumor bearing livers were harvested and fixed in 4% paraformaldehyde at 1 day after radiotherapy. The specimens were dehydrated in graded ethanol, embedded in paraffin, and cut into 5 μm-thick sections. The fixed sections were deparaffinized, hydrated according to a standard protocol, stained with hematoxylin&eosin (H&E) and immunohistologically stained against Ki-67 protein (Abcam, USA) respectively for microscopic observation. Apoptosis of the tumor cells in the mice after treatments was determined by the transferase-mediated dUTP nick end-labeling (TUNEL) assay according to the manufacturer's instructions 54.

### Statistical analysis

At least four independent experiments were carried out (n ≥ 4). Experimental data were expressed as Mean ± SD. The significant differences among groups were analyzed using one-way analysis of variance (ANOVA) in Origin software. The statistical significance was set at *P* < 0.05 level.

### Ethics Committee Approval

All animal experiments were carried out in accordance with the Institute of Laboratory Animal Resources guidelines. Ethical approval was granted by the Institutional Animal Care and Use Committee of Zhejiang University, China.

## Results and Discussion

### Characterization of multifunctional peptide modified ultrasmall Au NPs

**Figure 2:**
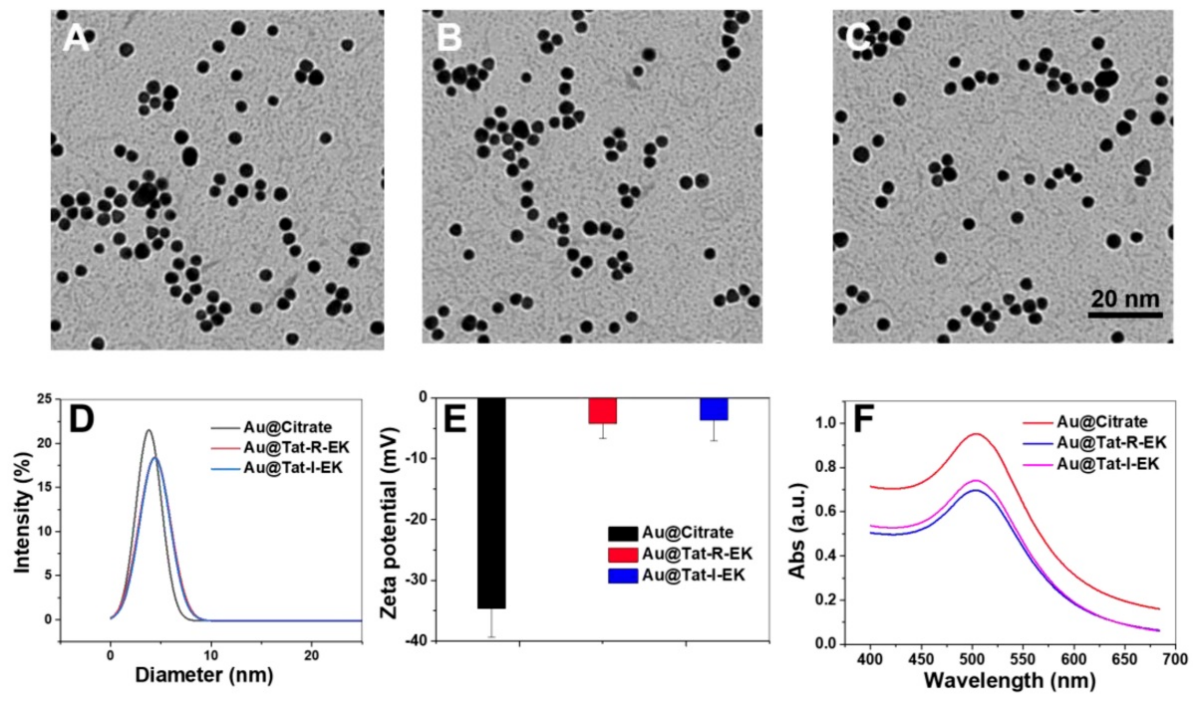
** Characterization of the physiochemical properties of Au NPs.** Representative TEM images of (A) original ultrasmall Au NPs, (B) ultrasmall Au@Tat-R-EK NPs, and (C) ultrasmall Au@Tat-I-EK NPs. (D) Histogram of hydrodynamic diameter and (E) surface zeta potential of ultrasmall Au NPs. (f) UV-vis spectra of ultrasmall Au NPs.

As shown in Figure 2A-B, highly monodisperse Au NPs with ultrasmall size (average diameters of 3.5 nm in dry state and 3.9 nm in aqueous solution respectively) were prepared based on previously reported method, taking the advantage of the addition of tannic acid to tune the kinetics of Au NP synthesis 51. The UV-vis spectra (Figure 2F) revealed that the localized surface plasmon resonance (LSPR) band of obtained Au NPs is peaking around 505 nm, which is in accordance with the feature of very small Au NPs 51.

With ultrasmall Au NPs in hand, multifunctional peptide Tat-R-EK and its non-responsive control Tat-I-EK were used for ligand exchange to modify their surface. The peptide was immobilized via the Au-S bonds between gold and thiol groups from cysteine. As shown in Figure S1, the Tat-R-EK peptide content on Au NPs increased with the feeding ratio and reached a plateau when the peptide concentration is higher than 50 μg/mL. Therefore, this peptide concentration was used for following functional Au NPs preparation, while the feeding ratio in weight between peptide and Au NPs (100 μg/mL) is 1:2. The peptide layer consists of about 11% of the overall weight of obtained Au@Tat-R-EK NPs, reached peptide density of 0.24 molecules per nm^2^ on Au NPs.

As shown in Figure 2B-C, the peptide coated Au NPs retained the spherical morphology, highly uniformity and ultrasmall size, indicating that the surface modification process had no obvious impact on the structure of Au NPs. The average hydrodynamic diameter of peptide modified Au NPs increased to 4.9 nm (Figure 2D), which should attribute to the thicker hydration layer generated by peptide coating. The surface zeta potential of peptide coated Au NPs became more neutral (Figure 2E), compared to original highly negatively charged surface, suggesting again the successful surface modification where zwitterionic peptide provide overall neutral charge. As shown in Figure 2F, the peptide modified Au NPs have generally the same LSPR peak of original Au NPs, indicating the similar ultrasmall size of modified Au NPs. All the results demonstrated that we have successfully prepared peptide coated ultrasmall Au NPs.

### Cathepsin B-responsive cell uptake and radiosensitivity *in vitro*

**Figure 3:**
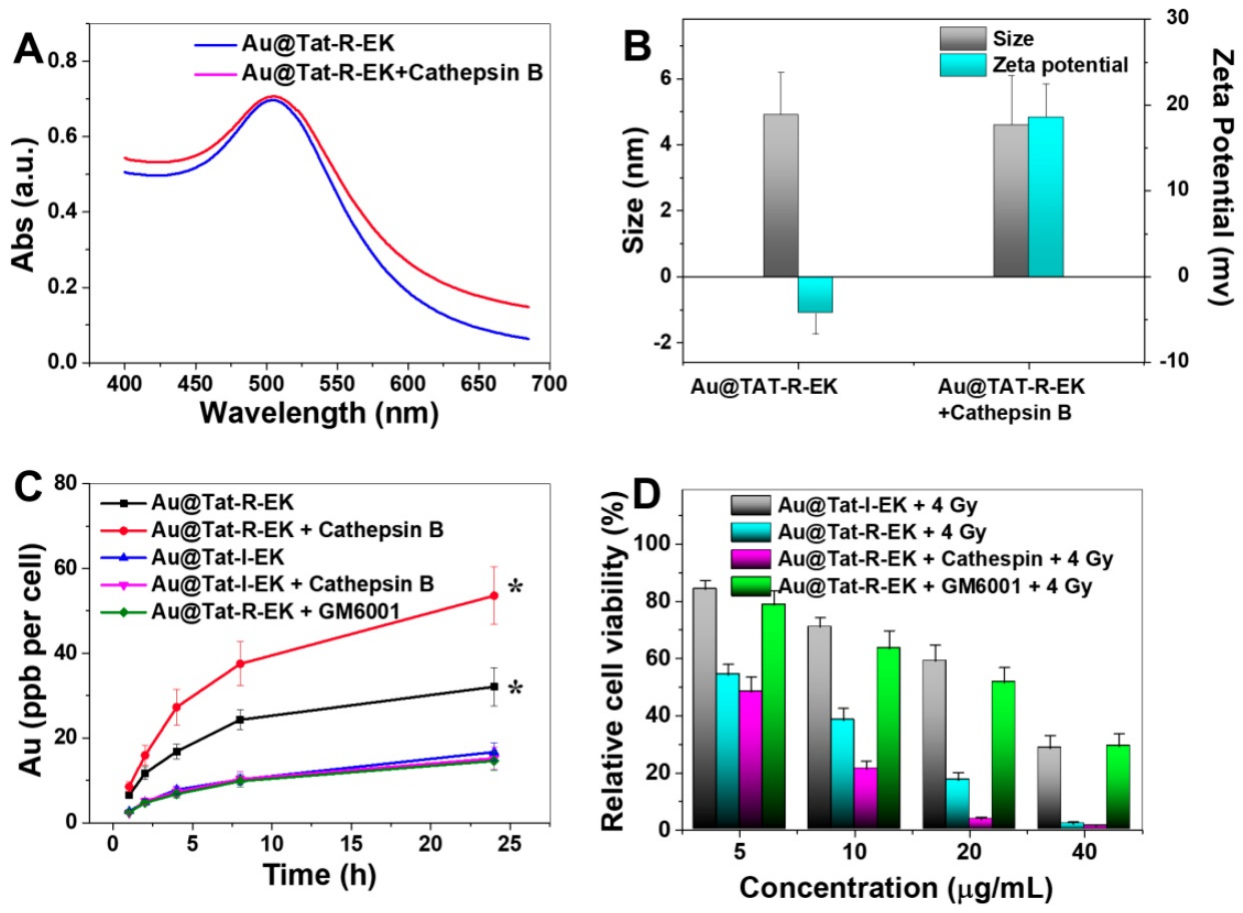
** Responsiveness, selective cell uptake and cytotoxicity of Au NPs.** (A) UV spectra, (B) hydrodynamic diameter and surface zeta potential of as-prepared ultrasmall Au@Tat-R-EK NPs and the ones treated with cathepsin B, respectively. (C) Plot of the gold concentration in LM3 cells as a function of incubation time that cells exposed to ultrasamll Au NPs (20 μg/mL, n=4). (D) Relative viablity of LM3 cells incubated with various concentrations of ultrasmall Au NPs for 24 h, and then treated with X-ray (4 Gy, n=4). * indicates significant difference at *P* < 0.05 level.

Cathepsin B is one of the lysosomal hydrolases, belongs to the cathepsin family. Overexpression of cathepsin B has been observed in malignant tumors and has been recognized to be closely correlated with tumor metastasis 45-47. Therefore, a short peptide sequence (GFLG) which can be selectively cleaved by cathepsin B was introduced into the multifunctional coating. We firstly studied the colloidal stability of responsive peptide modified Au NPs in a simulated biological environment. As shown in Figure S2, the Au@Tat-R-EK NPs had the same hydrodynamic diameter in PBS buffer at different pH values, indicating acidic tumor environment would not impair the stability of peptide coated Au NPs. Under the attack of cathepsin B, the outer amphiphilic peptide block would be cleaved and the inner positively-charged Tat sequence would be exposed. As a result (Figure 3B), the surface zeta potential of Au NPs shifted from negative to positive, indicating the successful responsiveness. The LSPR absorption at wavelengths over 550 nm slightly increased after the Au@Tat-R-EK NPs were treated with cathepsin B (Figure 3A). This can be attributed to slight aggregation of Au NPs after removing the protecting zwitterionic coating. However, the extent shall be very small because we could not observe a significant change of the hydrodynamic diameter (Figure 3B).

Then we studied the enzyme-responsive cell uptake and subsequent radiosensitivity *in vitro*. Both Au@Tat-I-EK NPs and Au@Tat-R-EK NPs have neglectable cytotoxicity at tested concentrations (Figure S3). As shown in Figure 3C, the cellular uptake of irresponsive ultrasmall Au@Tat-I-EK NPs was quite low within the whole experimental period with or without the additional cathepsin B, indicating the zwitterionic coating can provide good protection of Au NPs and efficiently prevent the cell uptake. At mean time, LM3 cells ingested much more Au@Tat-R-EK NPs in normal medium, reaching 2.3 times higher gold concentration inside cells after 24 h incubation than that of Au@Tat-I-EK group. More attractively, significantly higher uptake of Au@Tat-R-EK NPs by LM3 cells were found when we included additional cathepsin B (1 μg/mL) in the culture medium, reaching 3.6 times higher gold concentration inside cells at 24 h than that of Au@Tat-I-EK group. In the presence of GM6001, a cathepsin B inhibitor, the cellular uptake of Au@Tat-R-EK NPs was reduced to the level of Au@Tat-I-EK NPs. The results confirmed the cathepsin B responsive-dependent switch of cell penetrating ability of Au@Tat-R-EK NPs, suggesting their potential responsiveness to tumor microenvironment and thus selective uptake by tumor cells.

Encouraged by the promising cell uptake results, we then tested the radiation induced toxicity of Au@Tat-R-EK NPs against LM3 cells *in vitro*. Radiation alone has limited cytotoxicity: about 90% of cell viability was retained (Figure S4). As shown in Figure 3D, the radiation induced cytotoxicity gradually increased with feeding concentration in all Au NPs treated groups. This is understandable since usually the cell uptake amount of Au NPs is increased along with the feeding concentration, leading to stronger radiosensitivity 8. In general, the Au@Tat-I-EK NPs had limited contribution to enhanced cytotoxicity, due to the low cell uptake efficiency (Figure 3C). The radiosensitivity of Au@Tat-R-EK NPs was much stronger, leading to almost complete killing the cells at 40 μg/mL dosage. The highest radiation induced cytotoxicity was observed in Au@Tat-R-EK NPs + cathepsin B group, because of the highest intracellular gold concentration. It was worth noting that the radiation induced cytotoxicity of Au@Tat-R-EK NPs significantly reduced with the presence of cathepsin B inhibitor GM6001, proving again the key performance of enzyme-responsive feature.

**Figure 4:**
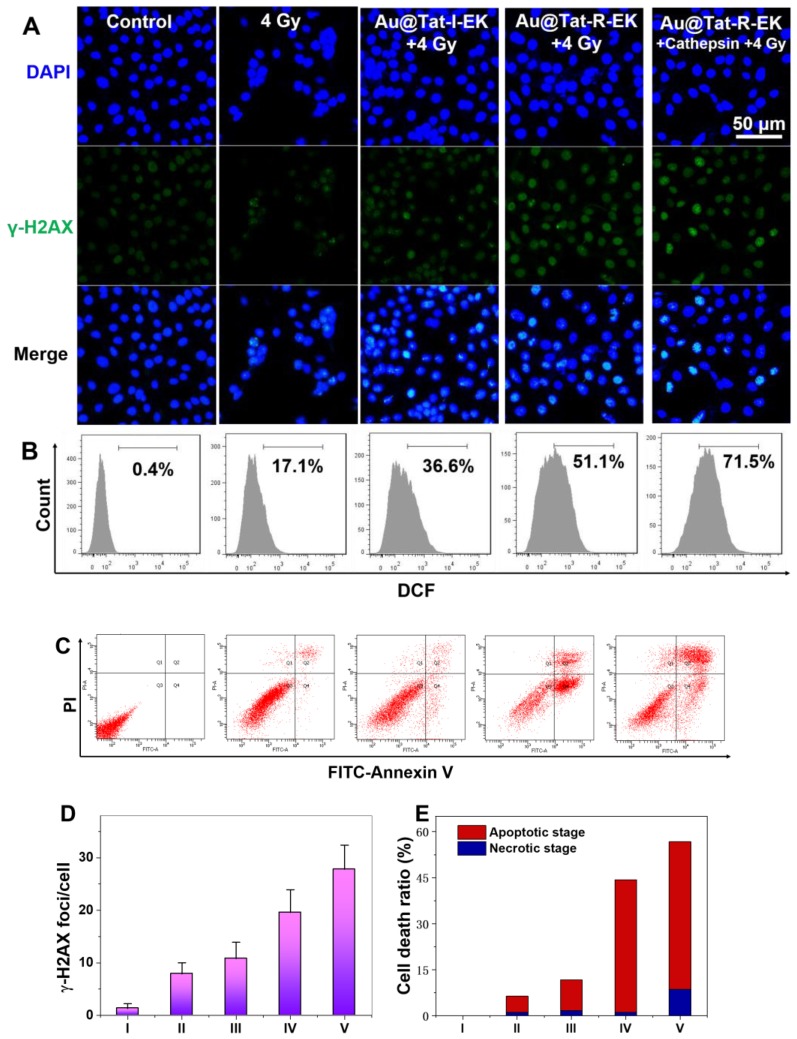
** Cytotoxicity mechanism of Au NPs in the presence of X-ray irradiation.** (A) γ-H2AX immunofluorescence, (B) Flowcytometry based intracellular ROS level analysis, (C) Flowcytometry based apoptosis analysis of LM3 cells after different treatments (n=4). Quantitative analysis of (D) γ-H2AX foci density, and (E) cell apoptosis and necrosis ratio in each treatment group. I: Control; II: 4 Gy; III: Au@Tat-I-EK + 4 Gy; IV: Au@Tat-R-EK + 4 Gy; V: Au@Tat-R-EK + Cathepsin + 4 Gy. The concentration of ultrasmall Au NPs is 20 μg/mL. Untreated cells were used as control.

Next we tried to have a closer look of the potential mechanism of the Au NPs-sensitized radiotherapy. It is well acknowledged that radiation-induced toxicities are derived from the intracellular DNA damage, typically the breakage of double-strand structure. γ-H2AX protein has been frequently reported as a sensitive indicator to evaluate the double-strand breakage. Figure 4A, D indicated slight enhancement of γ-H2AX foci for the cells irradiated with X-ray alone, while the average number of γ-H2AX foci increased to 7.9. After treated with Au NPs and X-ray, the number of γ-H2AX foci increased to 10.8 and 19.6 for the cells in Au@Tat-I-EK group and Au@Tat-R-EK group, respectively. The average number of γ-H2AX foci further increased to 27.8 in Au@Tat-R-EK group, with the presence of additianal cathepsin B in the medium, attributed to the faster exposure of cell penetrating peptide and thus enhanced cell uptake of Au NPs. The addition of GM6001 could inhibit the activity of cathepsin B and reduced the number of γ-H2AX foci to 13.5 (Figure S5).

Upon X-ray irradiation, the overexpressed ROS is responsible for the indirect DNA damage, lipid peroxidation, and protein oxidation, which are believed to be closely related with cell death. Therefore, we quantified the intracellular ROS levels after various treatments by flowcytometry assay using DCFH-DA as the intracellular ROS indicator. As shown in Figure 4B, a small proportion of cells (17.1%) overexpressed ROS was found upon merely X-ray irradiation. Addition of Au@Tat-I-EK NPs and Au@Tat-R-EK NPs before the radiotherapy enhanced the population of ROS positive cells to 36.6% and 51.1% respectively. With the help of additional cathepsin B, the ROS production inside cells was further augmented. In contrast, the reduction of cathepsin B activity could effectively suppress ROS generation (Figure S6).

With the DNA damage and ROS over-generation, cells will eventually become dead. Annexin V-FITC/PI assay was used to distinguish cells in different apoptotic phases using flow cytometry, which were identified as viable, early apoptotic, late apoptotic, and necrotic cells, respectively (Figure 4C, E). For the cells treated with nonresponsive Au@Tat-I-EK NPs and X-ray, the ratios of the apoptotic and necrotic cells were 10.1%, and 1.7%, respectively, similar to those of treated with merely X-ray. For the responsive Au@Tat-R-EK NPs and X-ray treatment, the ratio of the apoptotic cells increased to 43.1%. The populations of the apoptotic and necrotic cells were increased to 48.1 and 8.6% respectively, when the cells were treated with Au@Tat-R-EK NPs, additional cathepsin B and X-ray.

### *In vivo* Biocompatibility Evaluation of ultrasmall Au NPs

**Figure 5:**
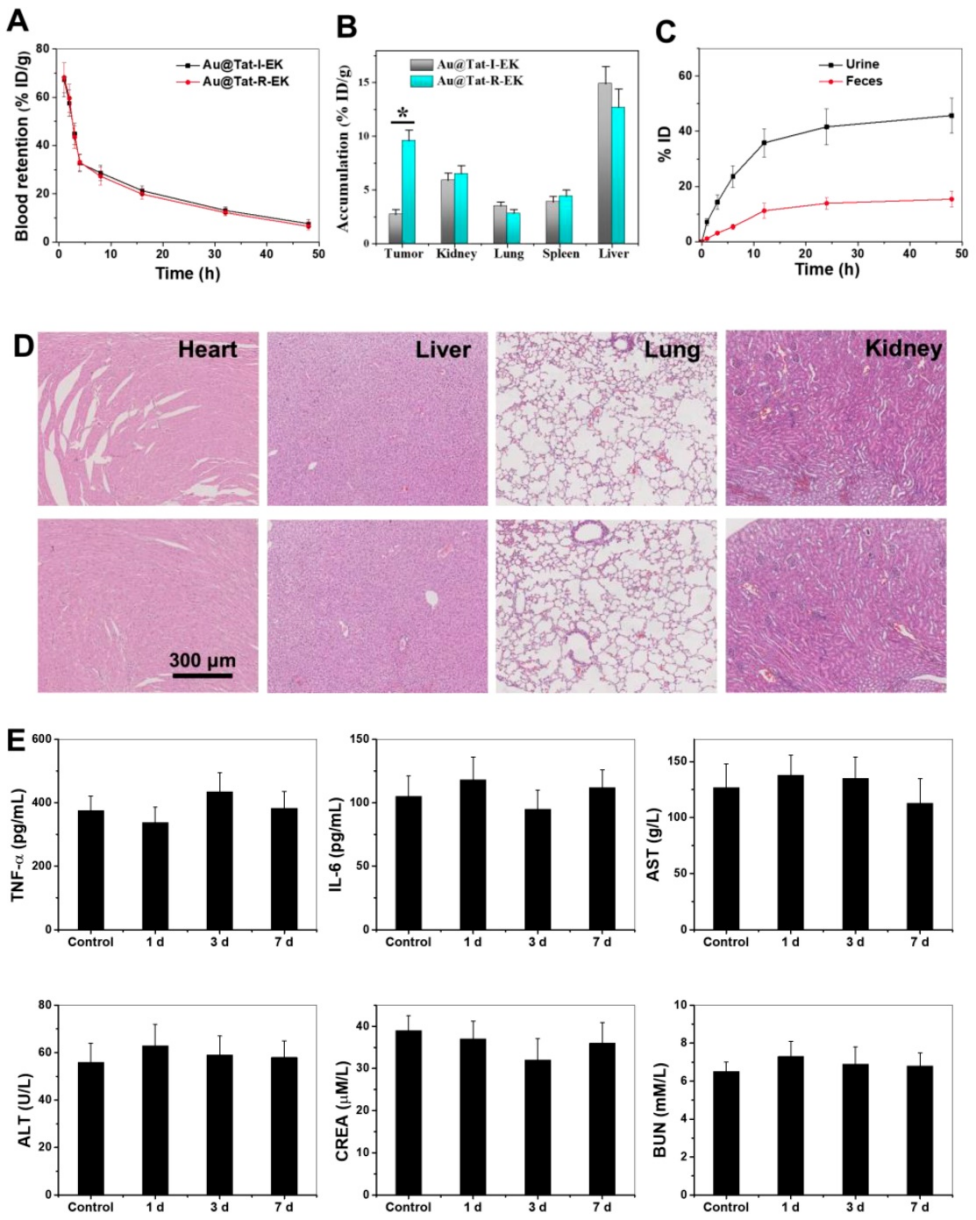
** Biodistribution, clearance and biocompatibility of Au NPs *in vivo*.** (A) The blood concentration of ultrasmall Au NPs as a function of time after intravenous injection. (B) The tissue distribution of ultrasmall Au NPs 24 h after intravenous injection. (C) Cumulative urine and feces excretion at different time points (n=4). * indicates significant difference between different groups within same time frame at *P* < 0.05 level. (D) Representative images of H&E stained major organs from mice treated with PBS and Au@Tat-R-EK NPs (50 mg/kg), respectively. Scale bar is 300 μm. (E) Blood test results of mice received Au@Tat-R-EK NPs i.v. injection once (n=4).

For a wide range of biomedical applications, ultrasmall Au NPs must exhibit biocompatibility* in vivo*. We evaluated the impacts of ultrasmall Au@Tat-R-EK NPs (50 mg/kg, two-fold greater than the concentration used for radiotherapy) on blood chemistry, inflammatory cytokine levels, and major organ histopathology in healthy mice to reveal their biocompatibility* in vivo*. As shown in Figure 5D and Figure S7, no necrosis, congestion, or hemorrhage was observed in the heart, liver, and lung at 1 day after intravenous injection of ultrasmall Au NPs.

The immunogenicity of ultrasmall Au NPs was then tested in terms of the serum concentrations of IL-6 and TNF-α at 24 h after intravenous injection. As shown in Figure 5E, the serum levels of IL-6 and TNF-α in the Au@Tat-R-EK NPs-treated group were identical to the levels in the control group (*P >* 0.05), indicating that ultrasmall Au NPs would not trigger obvious immune responses *in vivo* at the tested concentration. Additionally, the serum biochemistry analysis results (Figure 5E) showed that serum concentrations of liver function indicators (AST and ALT) and kidney function indicators (BUN and CRE) in the ultrasmall Au NPs-treated group were similar to those in the control group (*P >* 0.05), revealing good biocompatibility in the liver and kidney. Moreover, the results of complete blood panel analysis (Figure 5E and Figure S8-9) showed no obvious differences in the hematology of the Au@Tat-R-EK NPs-treated group when compared to that of the control group (*P >* 0.05). All the results confirmed that the synthesized ultrasmall Au@Tat-R-EK NPs exhibited negligible toxicity* in vivo*, suggesting potential usage in biomedical applications.

### Pharmacokinetics, Biodistribution, and Clearance of ultrasmall Au NPs in Mice

It is essential to understand the blood circulation, biodistribution, and organ clearance profiles of the nanomaterials before discussing their effects on diseases 55. An orthotopic transplanted liver tumor was generated on nude mice by injection of LM3 cells into the liver. The time-dependent blood circulation profiles of both ultrasmall Au NPs in Figure 5A demonstrated a classical two-compartment pharmacokinetic model. The blood clearance of Au@Tat-I-EK and Au@Tat-R-EK NPs were 2.17 h and 2.01 h, respectively. The biodistribution of Au NPs in the major organs of tumor bearing mice at 24 h post-injection was detected by ICP-MS. As shown in Figure 5B, the tumor in Au@Tat-R-EK group exhibited much higher dosage of Au NPs (9.6 ID%·g^-1^) than the one in Au@Tat-I-EK group (2.7 ID%·g^-1^). In contrast, the accumulation of both Au NPs in the heart, liver, and lung were quite similar. The accumulation of Au@Tat-R-EK NPs in the tumors should be attributed to the responsive surface shift in the tumor microenvironment. Moreover, time-dependent accumulation of ultrasmall Au NPs in the urine revealed that renal clearance was the main excretion pathway for these particles, rather than intestinal excretion (Figure 5C). Previous studies showed that the size threshold of the glomerular basement membrane (GBM) was approximately 5.5 nm, and nanomaterials with a diameter less than 5.5 nm could be effectively cleared from the blood to the renal tubules through the GBM 56. Approximately 62% of Au NPs could be excreted within 48 h post-injection (45% through the kidneys and 17% through the intestine), suggesting relatively rapid clearance and potentially low long-term toxicity of the ultrasmall Au@Tat-R-EK NPs.

### Radiation therapy *in vivo*

**Figure 6:**
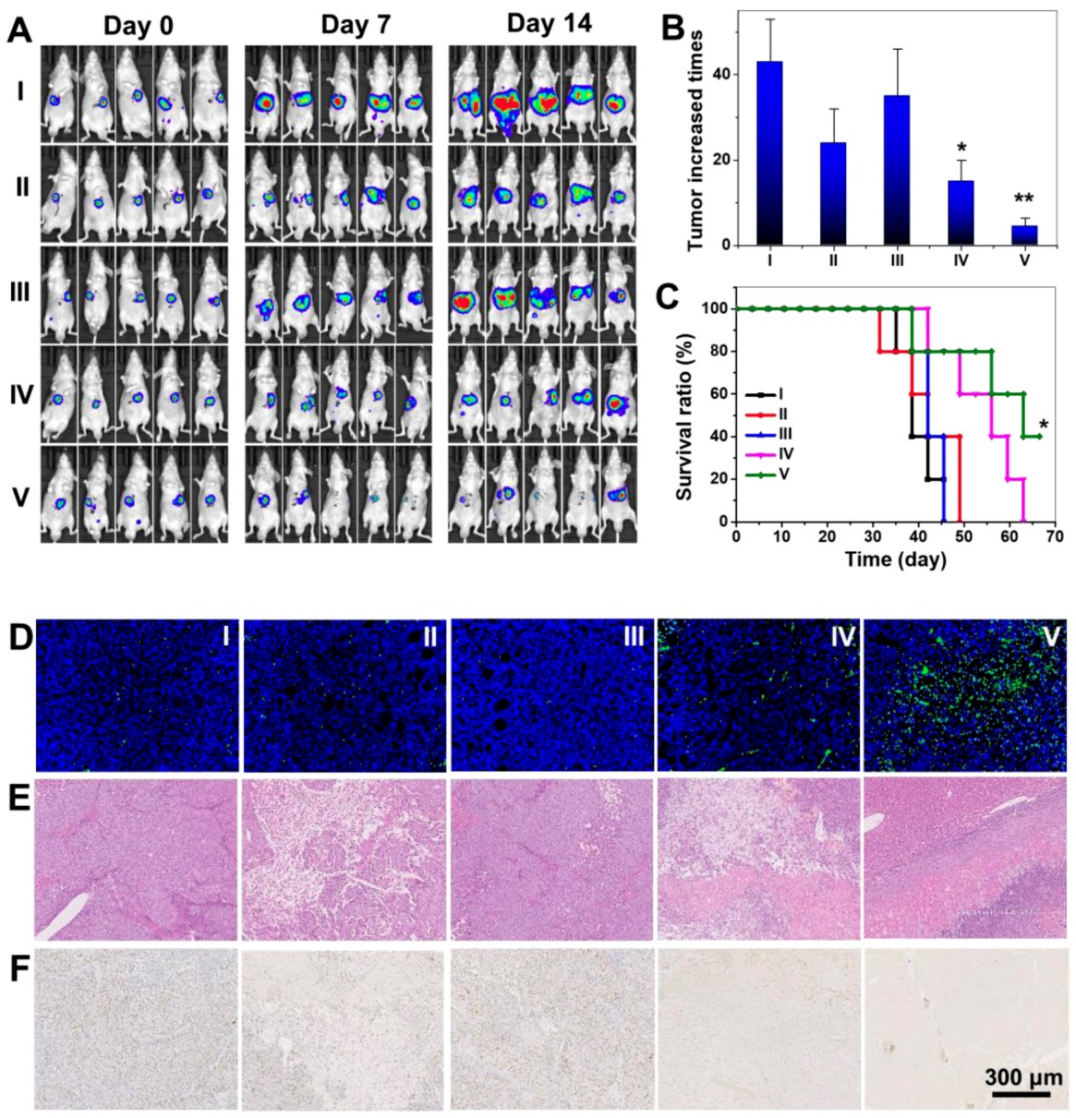
** Radiotherapy of Au NPs on orthotopic liver tumor.** (A) Fluorescent images of luciferase transfected LM3 Orthotopic liver tumor bearing mice. (B) The relative tumor weight increase fold at day 14 in different groups. (C) Survival rate of the mice bearing LM3 tumors after different treatments. (D) TUNEL, (E) H&E, and (F) Ki67 analyses of tumor tissues after various treatments. Scale bar is 300 μm. I: Control; II: 6 Gy; III: Au@Tat-R-EK; IV: Au@Tat-I-EK + 6 Gy; V: Au@Tat-R-EK + 6 Gy. The dosages of Au NPs and X-ray are 25 mg/kg and 6 Gy, respectively. * and ** indicate significant difference at *P* < 0.05 and *P* < 0.01 level, respectively.

With encouraging *in vitro* radio-sensitizing performance, biocompatibility and microenvironment-responsive tumor accumulation *in vivo*, we further performed tumor therapeutic evaluations on orthotopic transplanted LM3 liver tumor bearing nude mice. Mice were divided into five groups and treated with PBS, Au@Tat-I-EK NPs, and Au@Tat-R-EK NPs, followed by X-ray irradiation (6 Gy) 24 h post injection (Figure 6A). The body weight of mice in X-ray treated group decreased about 10% compared to untreated control group, indicating the overall toxicity induced by radiation (Figure S10). No obvious discrepancy was found between Au NPs + X-ray groups and merely X-ray group, indicating no substantial pathological damage was induced by Au NPs. The tumor volume was tracked by luciferase based fluorescent *in vivo* imaging assay (Figure 6A) and confirmed after sacrificed mice at day 14 (Figure S11). The tumors in control group and Au@Tat-R-EK NPs group showed fierce growth and reached 43 and 35 times than the original tumor volume at day 14 (Figure 6B). X-ray irradiation slightly relieved the exaggerating growth of the tumor (24 times of the original volume at day 14), which was attributed to the X-ray induced tissue damage. Treatments of both Au@Tat-I-EK NPs + X-ray and Au@Tat-R-EK NPs + X-ray augmented the radiotherapeutic performance. With higher tumor accumulations of microenvironment-responsive ultrasmall Au NPs, the Au@Tat-R-EK NPs + X-ray group had the slowest tumor growth with only 4.5 times tumor volume expansion than the original one at day 14. The tumor volume suppression rate reached 65% and 90% by Au@Tat-I-EK NPs and Au@Tat-R-EK NPs mediated radiotherapy respectively. As shown in Figure 6C, the Au@Tat-R-EK NPs + 6 Gy treatment can significantly prolong the survival time of mice (*P* < 0.05), compared to other groups.

The excellent anti-tumor efficacy of Au@Tat-R-EK NPs + X-ray was further verified by H&E, cell damage marker TUNEL, and cellular proliferation marker Ki-67 staining (Figure 6D-F), which revealed pathological changes in the tumor sections for the mice received different treatments. Negligible apoptosis/necrosis and high cell proliferation were observed in the tumor sites from the groups administrated with PBS, or Au@Tat-R-EK NPs, as evidenced by the intact nuclei morphology and high Ki-67 protein expression. High level of cell damage was observed for the mice treated with X-ray and Au@Tat-I-EK + X-ray because of their moderate anti-tumor ability. For the mice administrated with Au@Tat-R-EK NPs + X-ray, H&E staining revealed the fewest tumor cells and the most serious necrosis in tumor tissue. TUNEL and Ki-67 stainings further verified that the formulation of Au@Tat-R-EK NPs + X-ray led to the highest level of apoptosis and the lowest level of cell proliferation compared with the other treatments. Compared with conventional radiotherapy, these characterizations firmly demonstrated that Au@Tat-R-EK NPs were potent sensitizers for highly efficient radiotherapy of cancer, exhibiting enhanced therapeutic outcomes.

Au NPs are promising radiosensitizers due to their high atomic number and excellent biocompatibility without X-ray irradiation. Ultrasmall Au NPs or even smaller Au nanoclusters consisting only about a dozen of gold atoms have the unique advantage that they can be rapidly cleared through urea. Xie and Zhang et al. are the pioneers who prepared extremely small gold clusters and demonstrated their excellent potential in tumor radio-therapy 12, 57-59. However, relatively larger nanoparticles have better performance in tumor tissue accumulation due to EPR effect and more efficiently internalization by tumor cells 38-40. For example, Tsourkas et al. loaded ultrasmall Au NPs into polymeric micelles to achieve better tumor accumulation and enhanced radiosensitivity 60. Zhou et al. prepared Au NPs doped ROS responsive polymeric nanovesicles for controlled drug delivery and concurrent chemotherapy and radiation-therapy 61. However, the relatively larger sizes of the micelle and vesicle are not ideal for rapid renal clearance. Therefore, programmed aggregation strategies were developed to achieve a balance between rapid renal clearance and efficient tumor targeting. For example, gold nanoparticles can be programmed to assemble in tumor microenvironment and increase their anti-tumor efficacy 62. In current study, we develop a novel tri-block peptide to cover ultrasmall Au NPs for programmed tumor accumulation and cell internalization to enhance their radiosensitivity. The as-prepared ultrasmall Au@Tat-R-EK NPs are able to respond to overexpressed cathepsin B in tumor microenvironment, leading to site-specific enhancement of tumor cell uptake and subsequently effective DNA damage upon X-ray irradiation. Further *in vivo* experiments verified the satisfactory biocompatibility and tumor radiotherapy outcome against orthotopic LM3 liver tumors.

## Conclusions

In this study, multifunctional peptide modified ultrasmall Au NPs were prepared. The Au NPs exhibited enough colloidal stability and cathepsin B-responsive surface change, leading to selectively uptake by cancer cells *in vitro* and accumulation to tumor sites *in vivo*. Combined with X-ray irradiation, the responsive Au NPs demonstrated superior radio-sensitizing cytotoxicity *in vitro* and therapeutic outcome on mouse liver cancer *in vivo*. The ultrasmall size enables rapid clearance of the Au NPs, guarantees the biocompatibility *in vivo* for potential clinical applications.

Some obstacles faced by the Au NPs-based radiotherapy were effective solved through rational design of the nanomedicine, such as short circulation half-life, non-specifc distribution, fast clearance, and low radio-sensitizing effect. This work provides new insight in designing tumor microenvironment-responsive nanomedicine in cancer radiotherapy.

## Supplementary Material

Supplementary figures.

## Abbreviations

DAPI: 4',6-diamidino-2-phenylindole
DCF: fluorescent 2',7'-dichlorfluorescein
DLS: dynamic light scattering
DMEM: Dulbecco's modified eagle media
EPR: enhanced permeability and retention
FBS: fetal bovine serum
GBM: glomerular basement membrane
H&E: hematoxylin and eosin
HIV-1: human immunodeficiency virus-1
IL-6: interleukin-6
LSPR: localized surface plasmon resonance
MicroBCA: 2',7'-dichlorodihydrofluorescein diacetate
MTT: Methylthiazoletatrezolium
PI: propidium iodide
RES: reticulo-endothelial system
ROS: reactive oxygen species
TNF-α: tumor necrosis factor alpha
TUNEL: transferase-mediated dUTP nick end-labeling
