# Supplementary material for: Tumor microenvironment-responsive multifunctional peptide coated ultrasmall gold nanoparticles and their application in cancer radiotherapy
Source: Theranostics. 2020 Apr 6;10(12):5195–208. doi: 10.7150/thno.45017 (PMC7196283; doi:10.7150/thno.45017)
Supplement: Supplementary file 1 — Supplementary figures. [file thnov10p5195s1.pdf]

Supporting Information

Tumor microenvironment-responsive multifunctional peptide coated ultrasmall gold nanoparticles and their application in cancer radiotherapy

Yuan Ding[1,3,4,5,6#], Zhongquan Sun[1,3,4,5,6#], Zongrui Tong[2,3#], Sitong Zhang[1,3,4,5,6], Jie Min[1,3,4,5,6,8], Qianhui Xu[1,3,4,5,6], Liuzhi Zhou[1,3,4,5,6], Zhengwei Mao[2,3*], Haibing Xia[7*], Weilin Wang[1,3,4,5*]

[1] Department of Hepatobiliary and Pancreatic Surgery, the Second Affiliated Hospital, Zhejiang University School of Medicine, Hangzhou, Zhejiang 310009;

[2] MOE Key Laboratory of Macromolecular Synthesis and Functionalization, Department of Polymer Science and Engineering, Zhejiang University, Hangzhou 310027, China

[3] Key Laboratory of Precision Diagnosis and Treatment for Hepatobiliary and Pancreatic Tumor of Zhejiang Province, Hangzhou, Zhejiang 310009;

[4] Research Center of Diagnosis and Treatment Technology for Hepatocellular Carcinoma of Zhejiang Province, Clinical Research Center of Hepatobiliary and Pancreatic Diseases of Zhejiang Province, Hangzhou, Zhejiang 310009.

[5] Clinical Medicine Innovation Center of Precision Diagnosis and Treatment for Hepatobiliary and Pancreatic Disease of Zhejiang University, Hangzhou, Zhejiang;

[6] Clinical Research Center of Hepatobiliary and Pancreatic Diseases of Zhejiang Province, Hangzhou, Zhejiang 310009;

[7] State Key Laboratory of Crystal Materials, Shandong University, Jinan 250100;

[8] Department of Hepatobiliary and Pancreatic Surgery, the First Hospital of Jiaxing, Jiaxing, Zhejiang 330440.

# These authors contribute equally.

* Corresponding authors: wam@zju.edu.cn (W. W.); zwmao@zju.edu.cn (Z.

M.); hbxia@sdu.edu.cn (H. X.).

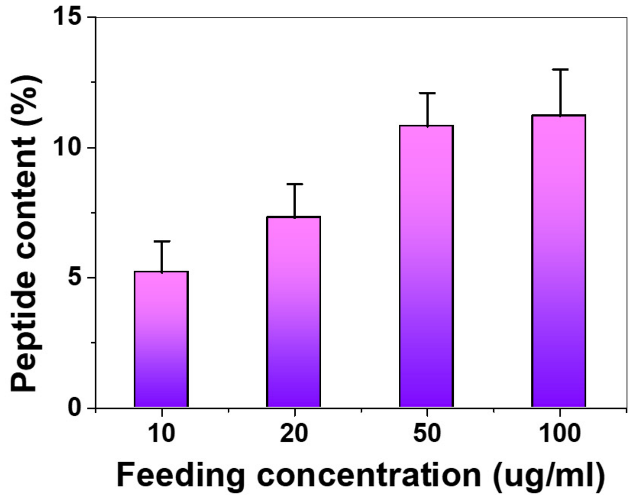

Figure S1. Peptide content on ultrasmall Au NPs surface as a function of the Tat-R-EK peptide feeding concentration. The concentration of Au NPs was 100 μg/mL.

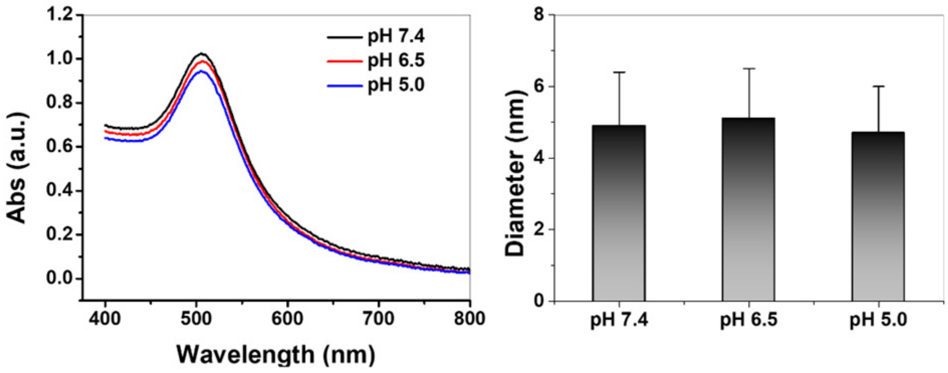

Figure S2. UV-vis spectra and hydrodynamic diameters of ultrasmall Au@Tat-R-EK NPs under different pH in PBS.

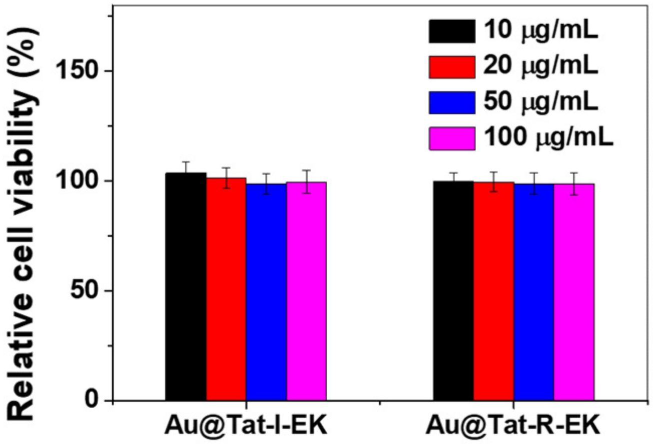

Figure S3. Relative cell viability of LM3 cells treated with ultrasmall Au NPs at various concentrations for 24 h. The untreated cells were used as control.

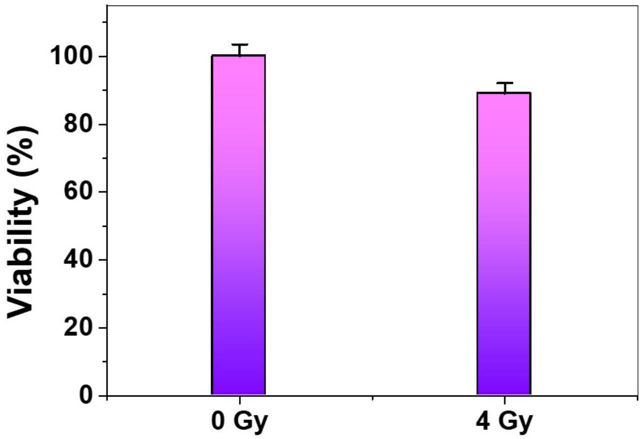

Figure S4. Relative cell viability of untreated LM3 cells (0 Gy) and cells received X-ray (4 Gy) alone.

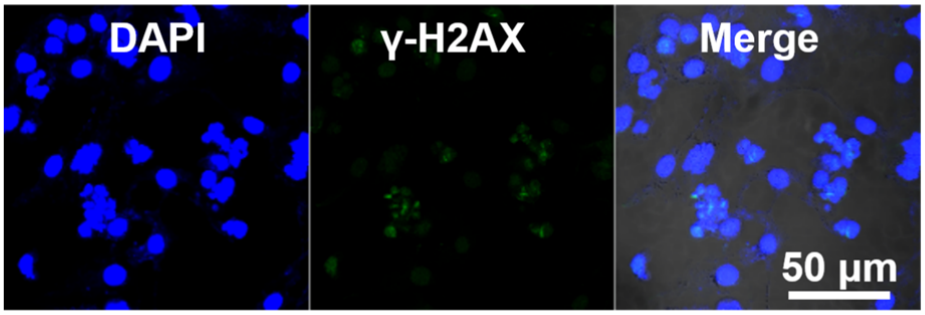

Figure S5. γ-H2AX immunofluorescence of LM3 cells treated with Au@Tat-R-EK NPs + GM6001, and 4 Gy radiation.

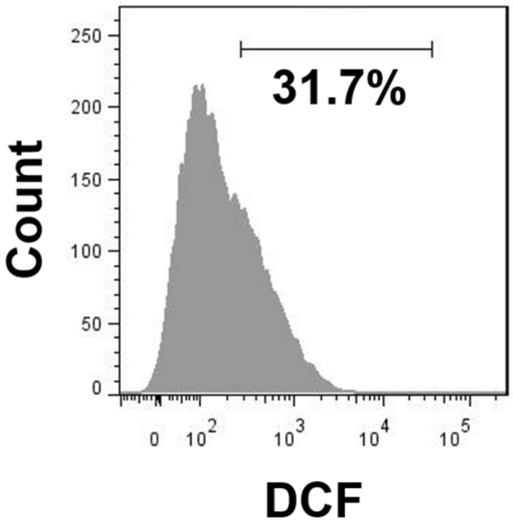

Figure S6. Flowcytometry based apoptosis analysis of LM3 cells treated with Au@Tat-R-EK + GM6001 + 4 Gy.

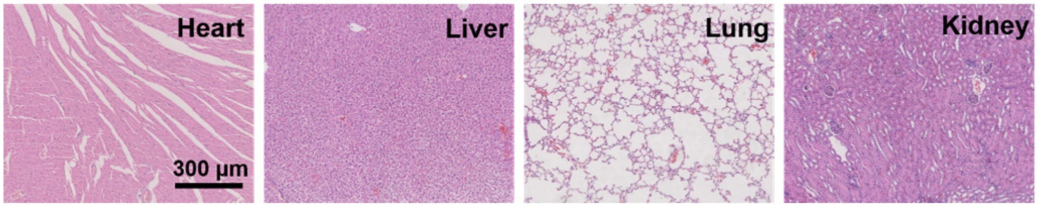

Figure S7. Representative images of H&E stained major organs from mice treated with Au@Tat-I-EK NPs (50 mg/kg).

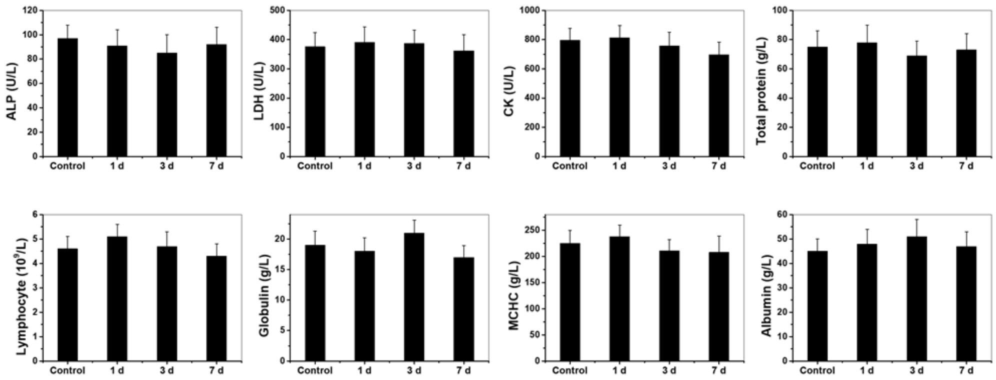

Figure S8. A part of blood test results of mice received Au@Tat-R-EK NPs i.v. injection once (n=4).

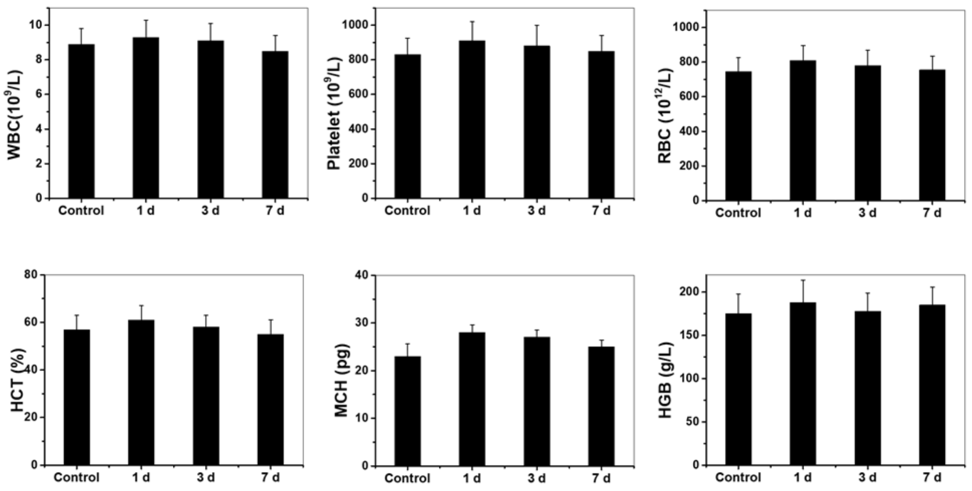

Figure S9. Another part of blood test results of mice received Au@Tat-R-EK NPs i.v. injection once (n=4).

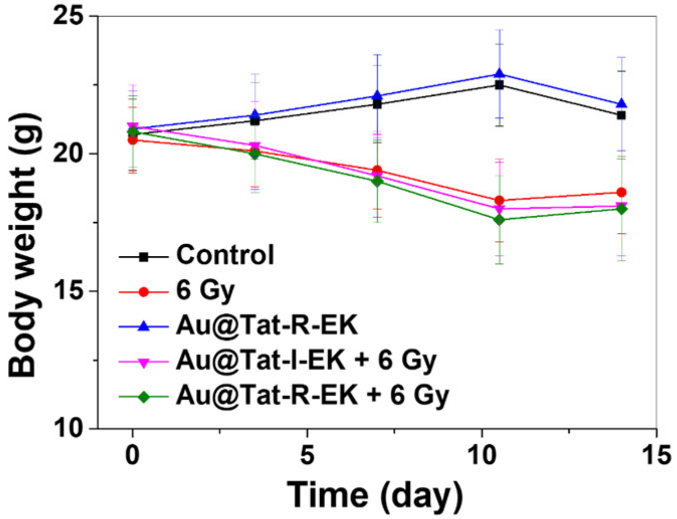

Figure S10. Body weight changes of the LM3 tumor-bearing mice treated with different formulations.

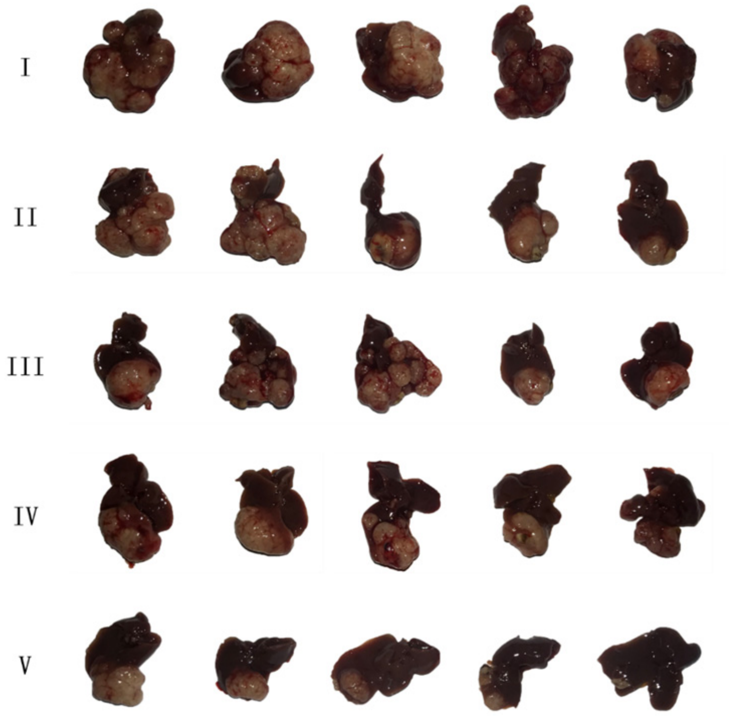

Figure S11. Photos of liver tumors after surgical excision. I: Control; II: 4 Gy; III: Au@Tat-R-EK; IV: Au@Tat-I-EK + 4 Gy; V: Au@Tat-R-EK + 4 Gy.
